# Upcycling of Waste Plastics into Carboxylic Acids for Biodegradable Surfactants

**DOI:** 10.1002/anie.202517471

**Published:** 2025-10-17

**Authors:** Houqian Li, Brandon W. Tipton, Hesham Aboukeila, Enner A. Mendoza, Abdulrahman Alzailaie, Tianwei Yan, Clark R. Landis, Javen S. Weston, Brian P. Grady, George W. Huber

**Affiliations:** ^1^ Department of Chemical and Biological Engineering University of Wisconsin‐Madison Madison WI 53706 USA; ^2^ School of Sustainable Chemical Biological and Materials Engineering University of Oklahoma Norman OK 73019 USA; ^3^ Department of Chemistry University of Wisconsin‐Madison Madison WI 53706 USA; ^4^ Department of Chemical Engineering University of Tulsa Tulsa OK 74104 USA

**Keywords:** Biodegradable surfactants, Hydroformylation, Oxidation, Process development, Waste plastics

## Abstract

This work outlines a process for producing high‐purity (>95%) carboxylate surfactants from post‐consumer recycled high‐density polyethylene (PCR‐HDPE). The approach involves the thermal depolymerization of PCR‐HDPE via pyrolysis, followed by fractional distillation to isolate C9–C14 olefins. These olefins undergo hydroformylation using cobalt carbonyl catalysts to generate aldehydes, which are subsequently oxidized to carboxylic acids using Pinnick oxidation under mild aqueous‐phase conditions. Neutralization of the resulting carboxylic acids with sodium hydroxide produces plastic‐derived carboxylate surfactants (PDCs) in the form of sodium carboxylates. Subsequent purification steps ensure surfactant‐grade purity and enable accurate assessment of physicochemical properties. The resulting PDCs are evaluated for critical micelle concentration (CMC), foamability, surface tension reduction, and calcium ion tolerance, demonstrating competitive behavior with conventional anionic carboxylate surfactants. This route provides a sustainable alternative for surfactant production, reducing reliance on fossil‐derived feedstocks and valorizing plastic waste streams through chemical upcycling.

## Graphical abstract

Biodegradable surfactants have now been created from post‐consumer waste HDPE. High conversion (>80%) is maintained throughout all major process steps including pyrolysis, hydroformylation, and oxidation. The utility of the plastic‐derived carboxylate surfactant is confirmed via critical micelle concentration, surface tension, and foaming measurements.

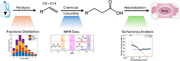

Waste plastics can be pyrolyzed at scale (i.e., 5–330 kilotons per year) to produce an olefin rich pyrolysis oil. Olefins are the central building blocks of the petroleum industry.^[^ ^1^ ^]^ These olefins can then undergo hydroformylation to form aldehydes, serving as intermediates for the synthesis of various petroleum chemicals such as alcohols, amines, and carboxylic acids.^[^ ^2^ ^]^ We have reported the production of aliphatic alcohols/diols and aliphatic amines/diamines from post‐consumer recycled high‐density polyethylene (PCR‐HDPE) via the combination of pyrolysis, hydroformylation, and hydrogenation or reductive amination previously.^[^ ^1^, ^2^, ^3^ ^]^ However, the production of high‐purity (>95%) carboxylic acids within a specific carbon range, which could potentially replace those currently derived from unsustainable resources, is yet to be demonstrated. C10–C15 (surfactant‐range) carboxylates are biodegradable surfactants generated by the neutralization, usually with sodium, of the corresponding carboxylic acids. The global surfactants market is expected to rise from $41.22 billion in 2021 to $57.81 billion in 2028.^[^ ^4^ ^]^ Carboxylic acids are typically derived from fossil‐based feedstocks via sequences involving petroleum cracking or Fischer–Tropsch synthesis of olefins, followed by hydroformylation and oxidation.^[^ ^5^ ^]^ In contrast, sodium carboxylates formed by the hydrolysis of vegetable oils or animal fats have served as surfactants for millennia.^[^ ^6^ ^]^ However, growing awareness of the environmental impacts associated with conventional surfactant production has prompted interest in more sustainable alternatives.^[^ ^7^ ^]^ In this work, we demonstrate that carboxylic acids can be sustainably generated from waste plastics through a series of reactions including pyrolysis, hydroformylation, and oxidation (Figure 1). Figure S1 includes the overall mass flow based on the experiments done in this manuscript. In the process outlined here, 27 kg of PDCs were produced from 100 kg of PCR‐HDPE. This process also forms light C1–C4 gases, relatively low molecular weight paraffins, and C11–C16 di‐acids all of which have large commercial markets with example applications being shown in Figure 1. Despite the tremendous work that has been performed to downcycle/recycle/upcycle waste plastics, converting real‐world waste plastics into products and characterizing their properties is still rare.^[^ ^8^, ^9^, ^10^ ^]^ Challenges to work with real plastics include the broad carbon distribution, the difficulties in controlling the oxidation process, the inorganics in the waste plastics and the purity requirements for surfactants.

**Figure 1:**
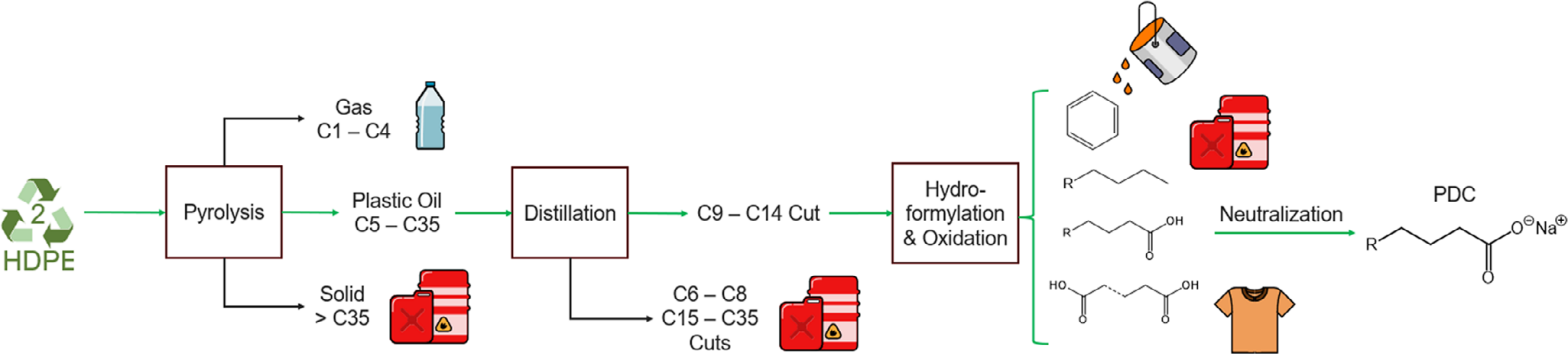
Process overview for plastic‐derived carboxylate surfactant (PDC) production.

The above‐mentioned challenges in producing plastic‐derived carboxylate surfactants (PDCs) are addressed in this publication by tuning the separation, chemistry, and purification processes. Alcohols could be sulfated to produce surfactants, but there has been increasing pressure for sulfate‐free formulations. As such, carboxylic acid groups were targeted here. A mixture of olefins, paraffins, and aromatics was generated from plastic pyrolysis. The plastic feedstock was PCR‐HDPE with the CHN(O)S results shown in Table S1. Typically, the liquid oils and solid waxes from pyrolysis are directed at downstream units such as reformers, hydrocrackers, or FCC units, where they are converted into fuels and feedstocks for petrochemicals.^[^ ^11^ ^]^ Fractional distillation of the plastic pyrolysis oil was performed at 195 °C and a vacuum of 50 mBar(g) to produce C9–C14 hydrocarbons using an in house glass distillation setup depicted in Figure S2. This resulted in a high ratio (86%) of these surfactant‐range hydrocarbons (Figure S3). A range of carbon numbers is acceptable for surfactant applications only being limited by the chain‐length compatibility effect.^[^ ^12^ ^]^ Previous research has shown that fractional distillation reduces metal concentrations, measured in ppm, by orders of magnitude.^[^ ^13^, ^14^ ^]^ These metals include iron, which, when mixed with the carbon monoxide stream introduced during hydroformylation can create iron carbonyls. Iron carbonyls can complex with noble metal catalysts poisoning the catalyst surface.^[^ ^15^ ^]^ As such their removal in this step is crucial for cobalt‐catalyzed hydroformylation. Inductively coupled plasma‐optical emission spectroscopy (ICP‐OES) confirms for this oil that fractional distillation led to a reduction in inorganic content (Tables S2–S4). Inorganic removal is crucial as without this step, deactivation of the hydroformylation catalyst becomes likely.

Hydroformylation converts the olefins into aldehydes which can be further functionalized. The generation of carboxylic acids from aldehydes requires a well‐controlled oxidation method to avoid side reactions.^[^ ^16^ ^]^ In addition, the properties of the obtained PDCs need to be tested to determine their suitability for surfactant applications. Due to the possibility for catalyst deactivation, experimentation began with model compounds such as 1‐hexene. Hydroformylation of 1‐hexene catalyzed by unmodified Co_2_(CO)_8_ led to the exclusive formation of aldehydes (Figure S4). In our previous work, we observed that aldehydes were also the main products for the hydroformylation of concentrated olefins (>60 wt.%) with comparable carbon lengths in light‐cut pyrolysis oil, up to 180 min of reaction time.^[^ ^2^ ^]^ Further hydrogenation of aldehydes into alcohols during hydroformylation was confirmed by NMR results (Figure S5). The presence of multiple peaks indicates the formation of aldehydes with varied structures, a result of regioselectivity and isomerization during hydroformylation (Figure S5a).^[^ ^17^ ^]^ Due to the presence of alcohols, additional carboxymethylation was necessary to convert these alcohols to carboxylic acids. Additionally, ester formation was observed, as evidenced by the ester carbon peak at 165 ppm (Figure S5).^[^ ^18^ ^]^ Hydroformylation of surfactant‐range pyrolysis oil was performed under the same conditions for 120 min to maximize aldehyde yield. The NMR spectrum suggests that olefins in the surfactant‐range plastic pyrolysis oil were completely converted, forming aldehydes, alcohols, and esters (Figure S5b). The molar percentages of aldehydes, alcohols, and esters in the hydroformylated oil were calculated using quantitative ^13^C NMR to be 60%, 33%, and 7%, respectively.

Pinnick oxidation (Scheme S1) was performed using hydroformylated dodecene and surfactant‐range hydroformylated oil. Pinnick oxidation was utilized due to its high selectivity in producing carboxylic acids from aldehydes under atmospheric temperature and pressure in aqueous conditions, as well as the use of relatively safe reagents, which reduces hazardous byproducts compared to oxidation methods that require stronger oxidants (e.g., KMnO_4_, O_2_).^[^ ^19^, ^20^ ^]^ The aldehydes in both model compound and plastic oil feedstocks were completely converted after Pinnick oxidation. This is evidenced by the appearance of carboxyl peaks in the alpha proton region in ^1^H NMR (Figure S6) and the disappearance of the peaks assigned to the formyl group in ^13^C NMR spectra (Figure S7). The non‐catalytic nature of this technique and the use of large amounts of solvents pose potential challenges for further advancing this oxidation procedure. Common practice is to use stoichiometric amounts of hazardous oxidants like potassium permanganate for aldehyde oxidation.^[^ ^21^ ^]^ However, oxidants such as molecular oxygen (from air) or hydrogen peroxide present a more sustainable oxidation route that could be worth exploring for future PDC production.^[^ ^22^ ^]^ GC‐MS identifies acid peaks after derivatization of the carboxylic acids to ensure no column degradation (Scheme S2). Branching due to the regioselectivity of hydroformylation is evidenced by the detection of multiple compounds with identical molecular formulas eluting at different retention times (Figure S8). The overlapping nature of the peaks in that chromatogram proves both the complexity of the oxidized oil samples and that there are still paraffins, alcohols, and esters in our product. These contaminants must be addressed before surfactancy testing. GC‐FID analysis confirms that the carbon distribution of carboxylic acids formed during the chemical conversion of pyrolysis oil falls within the desired surfactant range (Table S5).

Both alcohols and acids can be ethoxylated into nonionic surfactants (further sulfation can lead to ethoxylated sulfates) or can be sulfated directly into anionic surfactants.^[^ ^23^ ^]^ However, alcohols and fatty acids undergo ethoxylation at different rates, complicating surfactant synthesis of a mixture.^[^ ^24^ ^]^ On a large scale, distillation would be used to isolate the desired products from plastic pyrolysis oil, but this requires precise temperature control, which is challenging with our laboratory distillation system (Figure S2). We used the following approach to separate and purify the oxidized oil. We first tested the carboxymethylation of dodecanol as an average of surfactant range chain lengths to establish the formation of 2‐(dodecyloxy)‐acetic acid with the process for this being shown in Figure 2; a more detailed process description is shown in the Supporting Information. Sodium hydride (NaH) can be used to salt carboxylic acid and alcohol groups with near complete conversion.^[^ ^25^ ^]^ Following the addition of NaH, sodium monochloroacetate (SMCA) was used as a carboxymethylating agent. This adds a salted carboxylic acid group to the head of the alcohol while the PDCs do not react with SMCA. Multiple time scales and solvents were tested while developing this carboxymethylation and separation process with some of the results being shown in Table S6. The optimized conditions were then applied to the oxidized plastic oil over 96 h, and ^1^H NMR analysis (Figure S9) confirmed the near complete conversion of all alcohols to carboxylic acids and the recovery of the PDCs produced from Pinnick oxidation of hydroformylated surfactant‐range pyrolysis oil. This carboxymethylation step confers uniformity in surfactant properties and confirms assumptions underlying the interpretation of surfactant performance metrics. The presence of NaH does limit industrial utility. There are long standing safety concerns regarding NaH and its spontaneous ignition with atmospheric moisture.^[^ ^26^ ^]^ With the precise monitoring allowed at a lab scale along with proper quenching these concerns can be mitigated.

**Figure 2:**
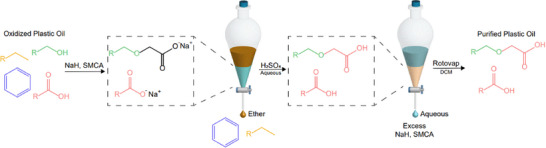
Process for carboxymethylating alcohols and purifying carboxylic acid groups in oil.

C10–C15 carboxylic acids were targeted as the final product considering the positive correlation between hydrocarbon chain length and CMC. A low CMC correlates to more effective emulsions at a given surfactant concentration.^[^ ^27^ ^]^ Previous green synthesis of surfactants have used biomass and PET plastic waste as feedstocks.^[^ ^24^, ^25^ ^]^ Here, we extend this list of possible feedstocks to include waste polyolefin plastics. Sodium decanoate (SDC) and sodium laurate (SLC) were used as reference surfactants as they have a carbon chain length of 10 and 12, respectively. Critical micelle concentration (CMC) values of the plastic‐derived carboxylate surfactants (PDCs) and the two reference surfactants were calculated from the plots of surface tension versus the logarithm of varying surfactant concentrations shown in Figure 3. In Figure 3a–c, the red low concentration line represents when the air–water interface becomes saturated with surfactants and additional molecules remain in solution as monomers. At higher concentrations, represented by the blue high concentration fitted line, the surface tension levels off as added surfactant no longer exists as monomers but instead aggregates into micelles. The intersection of these two linear fits defines the critical micelle concentration (CMC). The results were presented in weight percent as well as conventional molarity, due to the unknown molecular weight of PDC. The measured CMCs of SDC and SLC are in good agreement with literature values.^[^ ^28^ ^]^ PDCs showed a lower CMC than either reference surfactant, indicating that our surfactant has a higher surface activity. Wen et al. reported a CMC value of 0.1 wt% for sodium myristate (SMC), which is similar to the CMC measured for our PDC surfactant.^[^ ^29^ ^]^ This very low CMC value coupled with the analysis in Figure S10 suggests that some C14 carboxylate is present in the PDC mixture. Figure S10 also suggests that mixtures of homologous anionic surfactants, e.g. surfactants that have different chain lengths, have a CMC closer to the surfactant with the lower CMC (in this case the highest molecular weight) component in the sample.^[^ ^30^ ^]^ This result agrees with Figure S3a where weight percents of > C14 olefins are less prominent following fractional distillation.

**Figure 3:**
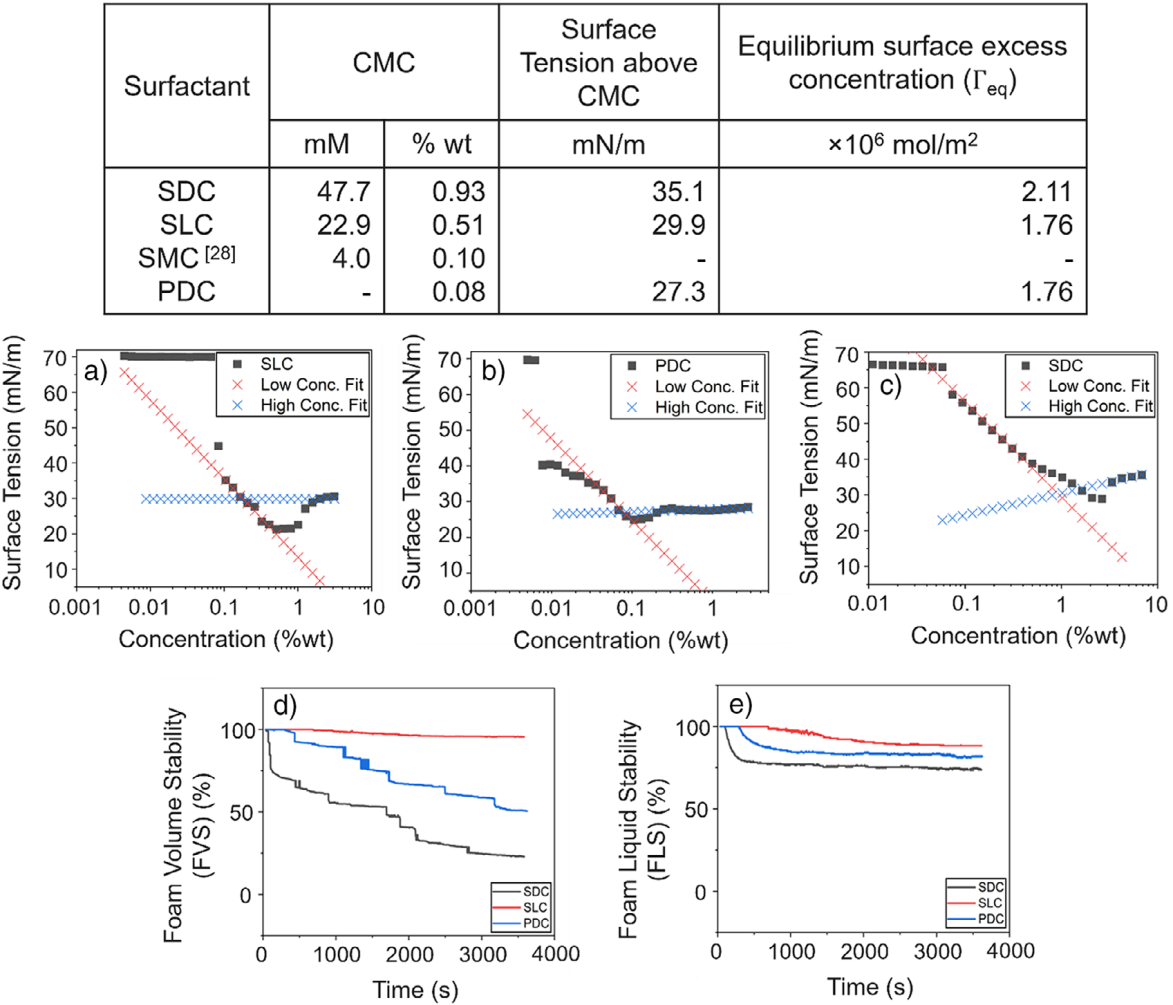
Graph of the surface tension versus concentration (%wt) for SLC a), PDC b) and SDC c). The intersection of the fitted lines shown was assigned as the CMC. Foaming property analysis including foam volume stability d) of the surfactant and foam liquid stability e).

Using a dynamic foam analyzer, the foam height decay over 3600 s was recorded, as illustrated in Figure S11. Ionic surfactants typically exhibit excellent foaming properties due to their strong electrostatic double‐layer effect and the pronounced Gibbs–Marangoni effect.^[^ ^31^ ^]^ All three surfactants reach foam stabilization at no longer than 50 s. PDC exhibited foam height higher than SDC and SLC initially; this height corresponds to something that foams well. However, SDC and PDC foam diminished more rapidly with time. The foam volume stability (FVS), foam liquid stability (FLS) and mean bubble area ($\bar{M}\bar{B}\bar{A}$) as a function of time are shown in Figure S11 with results from these graphs quantified in the table in Figure 4. Foam stability determines potential applications, e.g. lower stability is desirable in certain cleaning applications such as automatic dishwashing while higher stability is desired in other cleaning applications, such as dish cleaning by hand.^[^ ^32^ ^]^ PDC foam was less stable than SLC but more stable than SDC. Surfactants with longer alkyl chains usually exhibit higher foam stability.^[^ ^33^ ^]^ Photographs of the bubbles at different time intervals were also taken for visualization (Figure 4). FVS represents the percentage of foam volume retained compared to the maximum foam volume after foaming has stopped. FLS measures the foam's ability to retain liquid, reflecting its dryness.

**Figure 4:**
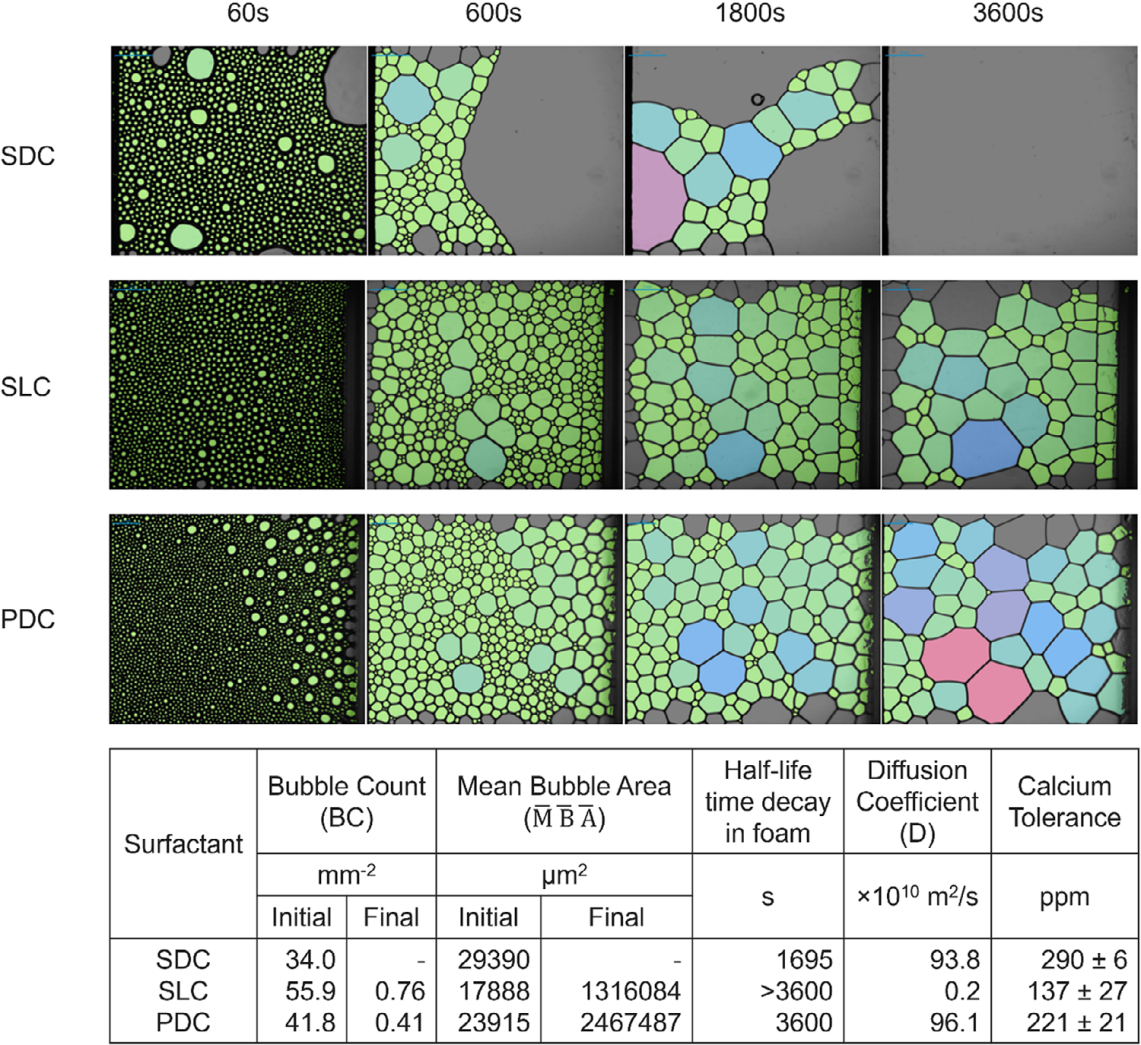
Microscopic measurements of SDC, SLC, and PDC at different time intervals at lens height of 55 mm along with the physicochemical surfactant characteristics. The different colors are labels automatically assigned by the image analysis software with no exact size correlation.

The diffusion coefficient (*D*) was calculated from the Ward & Toradi model (Equations S1 and S2) with the value for Γeq being shown in the table in Figure 3 and these coefficients are reported in Figure 4. Unlike CMC measurements, at short times, lower molecular weight components dominate diffusion constant measurements.^[^ ^34^ ^]^ The measured large diffusion constant, in conjunction with additional property data, suggests the coexistence of high molecular weight species and low molecular weight species (chain lengths lower than C10), indicative of a wide chain length distribution. Again, this was an expected result given the range of carbon numbers following fractional distillation. The diffusivity coefficient for PDC was similar with SDC but 2 orders of magnitude higher than SLC, which is clear evidence of a C10 or lower carbon number component in the mixture.

Finally, small‐angle X‐ray scattering (SAXS) was employed to investigate the nanoscale structure and self‐assembly of surfactants into micelles. SAXS results, provided in Figure S12, offer insight into micelle size, shape, and internal organization in solution. The results show that PDC has larger micelles compared to SDC and SLC which is consistent with PDC containing longer chain components. Detailed fitting of the SAXS data confirmed this assignment with parameters shown in (Table S7). The shape of all three surfactant micelles best fit as an oblate core‐shell‐ellipsoid with PDC having the largest size.

In conclusion, anionic surfactants were produced from PCR‐HDPE through sequential pyrolysis, fractional distillation, hydroformylation, oxidation, purification, and neutralization. Conditions for high conversions were found for all major process steps. Fractional distillation of pyrolysis oil allows for both inorganic removal and carbon‐range cuts suitable for surfactant synthesis. Hydroformylation of concentrated C9–C14 olefins (>50 wt%) leads to complete olefin conversion, forming primarily aldehydes alongside alcohols and esters as side products. Alcohol formation becomes more prominent with extended reaction times, as aldehydes are gradually hydrogenated under the reaction conditions. Carboxymethylation of these alcohols with SMCA provides an efficient route for the production of high purity carboxylic acid streams. This reaction is most commonly used in the polysaccharide industry to improve bioactivity.^[^ ^35^ ^]^ Still, improving the chemoselectivity of our hydroformylation system through catalyst selection or reaction time optimization makes our process less reliant on these additional reaction steps. Pinnick oxidation selectively converts aldehydes to acids under mild conditions (aqueous phase at room temperature), though its non‐catalytic nature and solvent use may limit scalability. This approach affords C10–C15 acids, yielding low‐CMC surfactants with favorable properties including foam stability and calcium tolerance. Our findings establish that polyolefin waste can be chemically upgraded into carboxylate surfactants with performance metrics such as CMC, foam stability, surface activity, and divalent ion tolerance that are comparable to those of conventional anionic surfactants derived from linear hydrocarbons.

## Supporting Information

The authors have cited additional references in the Supporting Information.^[^ ^2^, ^8^, ^13^, ^16^, ^36^, ^37^, ^38^, ^39^, ^40^, ^41^, ^42^, ^43^, ^44^ ^]^

## Conflict of Interests

H.L., C.R.L., and G.W.H. are inventors on US patent US20240158324A1 “Method for making aldehydes, alcohols, amides, and carboxylic acids from plastic pyrolysis oil”. G.W.H. has an ownership interest in Anellotech, which is commercializing the PlasTCat technology.

## Supporting information



Supporting Information

## Data Availability

The data that support the findings of this study are available in the supplementary material of this article.
