# Supplementary material for: Upcycling of Waste Plastics into Carboxylic Acids for Biodegradable Surfactants
Source: Angew Chem Int Ed Engl. 2025 Oct 17;64(50):e202517471. doi: 10.1002/anie.202517471 (PMC12684362; doi:10.1002/anie.202517471)
Supplement: Supplementary file 1 — Supporting Information [file ANIE-64-e202517471-s001.docx]

**Upcycling of Waste Plastics into Carboxylic Acids for Biodegradable Surfactants**

*Houqian Li ^1 ǂ^ *, Brandon W. Tipton ^1 ǂ^, Hesham Aboukeila ^2^, Enner Mendoza ^1^, Abdulrahman Alzailaie ^1^, Tianwei Yan ^1^, Clark R. Landis ^3^, Javen S. Weston ^4^, Brian P. Grady ^2^, George W. Huber ^1^**

^1^ Department of Chemical and Biological Engineering, University of Wisconsin-Madison; Madison, WI 53706 (USA)

^2^ School of Sustainable Chemical, Biological and Materials Engineering, University of Oklahoma, Norman, OK 73019 (USA)

^3^ Department of Chemistry, University of Wisconsin-Madison; Madison, WI 53706 (USA)

^4^ Department of Chemical Engineering, University of Tulsa, Tulsa, OK 74104 (USA)

*Corresponding authors: hqli@nmsu.edu, gwhuber@wisc.edu

ǂ: These authors contribute equally to this work.

**KEYWORDS**: Waste Plastics, Upcycling, Hydroformylation, Oxidation, Carboxylic Acids, Biodegradable Surfactants, Process Development

**Supplementary Materials**

**List of Chemicals**

Co(NO_3_)_2_·6H_2_O (239267), 1-dodecene (44148), Co_2_(CO)_8_ (34851), hydrogen peroxide (HX0635-3), potassium phosphate (PX1564-1), sodium sulfite (239321-500G), dichloromethane (676853-1L), magnesium sulfate (793612-500G), anhydrous tetrahydrofuran (401757-2L), sulfuric acid (339741-500ML), iodine (207772-100G), hexamethylphosphoramide (HMPA) (H11602-5G), and diethyl ether (673811-1L), all from Sigma Aldrich, sodium chlorite (RDCS0440-500B1, Ricca Chemical), hydrochloric acid (A144-212, ThermoFischer), sodium hydride (SC-253585A, ChemCruz), sodium monochloroacetate (SMCA) (404415000, Acros), sodium decanoate (TCI chemicals), sodium laurate (Acros Organics), calcium chloride (Sigma), magnesium chloride (Sigma-Aldrich), sodium hydroxide (Millipore), and syngas with CO/H_2_=1 from Airgas were used as purchased. Pyrolysis of the PCR-HDPE (MW:162,920, MN:27,292, obtained from an operational material recovery facility) was described in our previous work.^[1]^

**Experimental Methods with Results**

***Pyrolysis oil analysis and plastic contaminant removal***

Pyrolysis of plastic polyolefins generates products ranging from light gases like methane to heavy waxes (~C70)^[2]^, including aromatics, paraffins, olefins, and diolefins of varying chain lengths. Industrially, fractional distillation separates crude oil into fractions based on boiling points, including light gases (C1–C4), naphtha, kerosene, diesel, and gas oil.^[3]^ The C9 – C14 cut is used here to make plastic derived carboxylate surfactants (PDCs) with the process and mass balance being shown below in **Figure S1**.


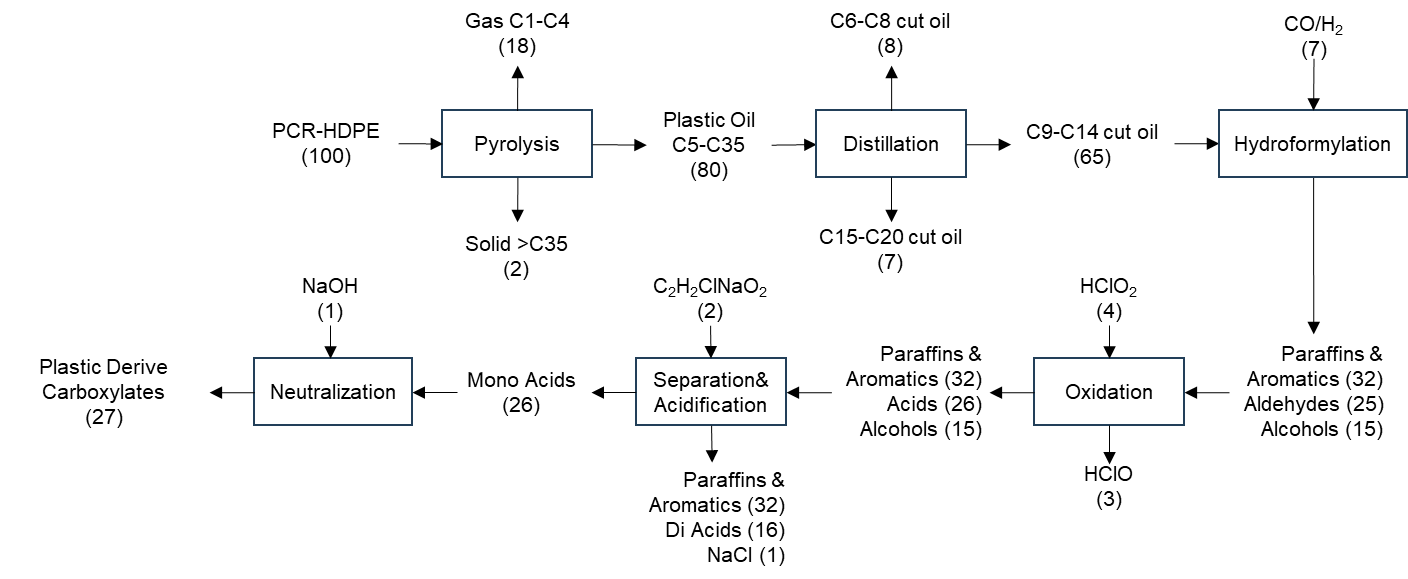


**Figure S1.** Proposed approach to produce surfactant-range carboxylic acids from waste polyolefins. Numbers in parentheses indicate the overall mass flow of materials.

The generated plastic oil from pyrolysis was fractionally distilled. A glass vacuum distillation setup (**Figure S2**) was used. A vacuum pressure of 50 mBar(g) was created using an MVP model 015-2 Pfeiffer vacuum pump. C9 and below hydrocarbons were distilled at approximately 50 °C. The silicone oil bath was then raised to 195 °C to separate the C9–C14 hydrocarbons, known here as “surfactant-range oil”, leaving C15 and above remaining in solution. To quantify the fractionally distilled pyrolysis oil a Shimadzu 2010 GC-FID with a 10 m Restek MXT-1HT column installed was used. Initially the oven was ramped to 350 ℃ and held for 5 min. Then the oven was heated to 415 ℃ with a ramp rate of 6 ℃/min and then this temperature was held for 6.15 min.


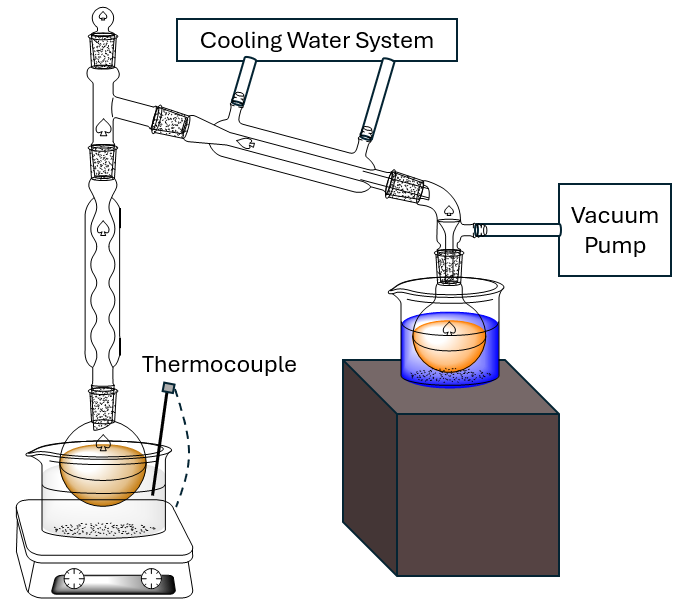


**Figure S2.** Plastic oil fractional distillation setup under vacuum.

Much higher purity is achievable in industry with more plates as demonstrated by using process modeling.^[2]^ The results of this distillation are shown below in **Figure S3**. The “Other” label, depicted in **Figure S3b**, accounts for aromatics and highly branched paraffins and olefins that are difficult to properly characterize. Fractional distillation also serves as a method of contaminant removal from PCR plastic waste.^[4]^


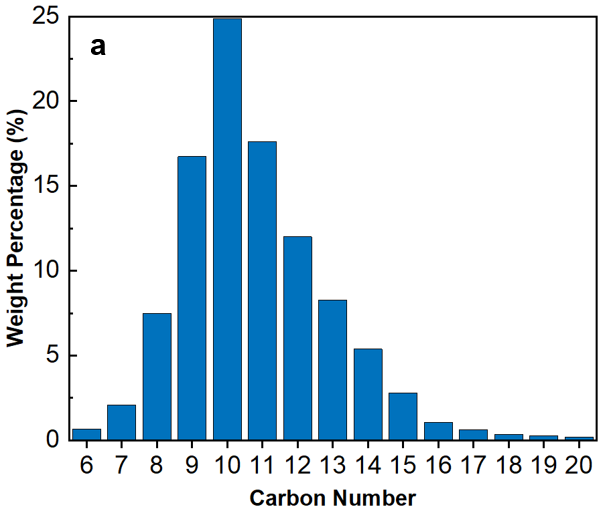

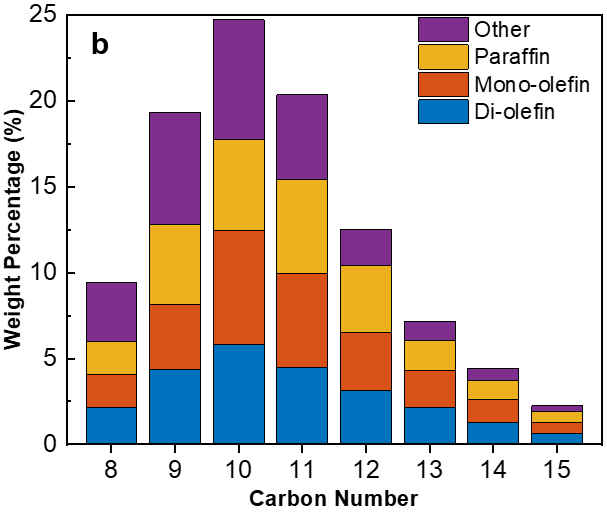


**Figure S3.** (a) The mass fraction distribution of the PCR-HDPE pyrolysis oil. (b) The chemical composition of the distilled fraction from PCR-HDPE plastic pyrolysis oil. “Other” includes aromatics, highly branched paraffins and olefins.

CHNS/O elemental analysis was conducted on the plastic feed material using a vario EL cube analyzer (Elementar, Germany) equipped with a thermal conductivity detector (TCD), and Infrared detector (IRD). Due to the instrument calibration constraints, the C, H, and S analyses were performed with a sample mass of 2.5 ±0.5 mg, whereas nitrogen, present minimally in polyolefin, required a larger sample mass of approximately 40 mg and was analyzed separately. Prior to analysis, samples were sealed in tin capsules and pressed to minimize trapped air. C, H, N, and S were measured using the combustion mode. The plastic used in this study was PCR-HDPE, containing elevated inorganic ash content. To determine the ash content, 10 g of the plastic feedstock was placed in an alumina boat and combusted in a muffle furnace at 550 °C for 2 hours. The weight difference was interpreted as the ash percentage with these results being shown in **Table S1**.

**Table S1**. Ultimate and proximate analyses of the plastic feedstock used in this study.

| **Analyte** | **Value (wt%)** |
| --- | --- |
| C | 85.10 ± 0.15 |
| H | 14.72 ± 0.07 |
| S | 0.22 ± 0.02 |
| N | 0.005 |
| Ash | 1.090 |

Plastic feedstocks, particularly post-consumer recyclate, have many trace elements present beyond the plastic polymer.^[2]^ To give examples, calcium is used in common plastic fillers and titanium is an ingredient in dyes. Inductively Coupled Plasma-Optical Emission Spectroscopy (ICP-OES) confirmed the initial presence of these contaminants in the PCR-HDPE feedstock tested here. Agilent 5800 Inductively Coupled Plasma-Optical Emission Spectroscopy (ICP-OES) was used to analyze the following elements concentration in both the PCR-HDPE plastic feed and the C9–C14 distilled oil: Ag, Al, B, Ba, Ca, Cd, Cr, Fe, Mg, Na, Pb, Sr, Ti, and Zn. These results, after calibration, are shown in **Table S2** which indicate a reduction in many of the major metal contaminants.

**Table S2**. The ICP-OES elemental analysis for the plastic feedstock and the distilled C9–C14 oil.

| **Element** | **PE plastic (ppmw)** | **C9-C14 oil (ppmw)** |
| --- | --- | --- |
| Ag | 0.3 | < LOD |
| Al | 464.0 | 46.10* |
| B | 221.0 | 82.10* |
| Ba | 53.3 | 0.21 |
| Ca | 2393.0 | < LOD |
| Cd | 123.0 | < LOD |
| Cr | 70.2 | < LOD |
| Fe | 242.0 | 5.01 |
| Mg | 85.7 | 3.46 |
| Na | 3443.0 | 289.00* |
| Pb | 305.0 | < LOD |
| Sr | 10.1 | 0.07 |
| Ti | 306.0 | 0.08 |
| Zn | 72.8 | 13.60 |
| * Qualitative |  |  |

Calibration standards (45.7, 11.5, 2.9, 0.7, and 0.2 ppmw) were prepared by mass dilution from ICP multi-element standard solution IV Certipur®; titanium was added separately at half the concentration of the other elements. A calibration blank (undigested) was used to establish the ICP-OES baseline. Each sample was analyzed in triplicate. Axial plasma viewing mode was used for all elements. Three wavelengths per element were evaluated, and the one with the highest intensity and minimal spectral interference (per element) was selected to report data. Limits of detection (LOD) and quantification (LOQ) were calculated based on multiplying the standard deviation of 30 replicate digested blank measurements by three and ten, respectively, see **Table S3.**

**Table S3**. Selected wavelengths for ICP-OES analysis and the corresponding limits of detection (LOD) and quantification (LOQ). Values were determined from 30 replicate reagent blanks.

| **Element** | **Wavelength (nm)** | **LOD (ppb)** | **LOQ (ppb)** |
| --- | --- | --- | --- |
| Ag | 328.068 | 0.84 | 2.81 |
| Al | 237.312 | 12.20 | 40.68 |
| B | 249.678 | 5.25 | 17.50 |
| Ba | 455.403 | 0.80 | 2.65 |
| Ca | 422.673 | 1.33 | 4.43 |
| Cd | 228.802 | 0.79 | 2.63 |
| Cr | 267.716 | 0.87 | 2.90 |
| Fe | 259.940 | 4.91 | 16.37 |
| Mg | 285.213 | 0.52 | 1.72 |
| Na | 588.995 | 21.34 | 71.14 |
| Pb | 220.353 | 20.56 | 68.53 |
| Sr | 421.552 | 0.44 | 1.45 |
| Ti | 334.941 | 31.10 | 103.66 |
| Zn | 213.857 | 1.13 | 3.77 |

Prior to ICP-OES analysis, approximately 250 mg of each sample was digested using concentrated nitric acid (70wt% HNO_3_) in a closed-chamber microwave digestion system (UltraWAVE, Italy). Detailed digestion parameters are provided in **Table S4**. The digested samples were diluted with MQ water (≥ 18.2 MΩ·cm) at a 1:9 ratio and filtered through a 0.45 um PTFE filter. A reagent blank (no sample) was digested under identical conditions.

**Table S4**. Microwave digestion parameters to prepare sample for ICP-OES analysis.

| **Parameter** | **Value** |
| --- | --- |
| Temperature (°C) | 260 |
| Initial N_2_ pressure (bar) | 40 |
| Ramp rate (°C/min) | 8 |
| Ramping time (min) | 30 |
| Hold time (min) | 20 |
| Power (W) | 1500 |

***Evaluation of chemical upcycling techniques***

Hydroformylation and removal of the Co catalyst were performed according to our previous experiments.^[1]^ Model compounds like 1-hexene were initially tested to confirm aldehyde formation, **Figure S4**. After this confirmation, 10 g of the C9–C14 pyrolysis oil was mixed with 1 g of Co_2_(CO)_8_ in a glove box, followed by diluting it to a 50 mL hexane solution. The mixture was then transferred to a 300 mL stainless steel Parr reactor, purged with the syngas thrice, pressurized to 10 Bar with the syngas, and then increased to 120 °C in 25 mins. Once the temperature reached 120 °C, the system was pressurized to 70 Bar with syngas and the clock time was set to 0 min.


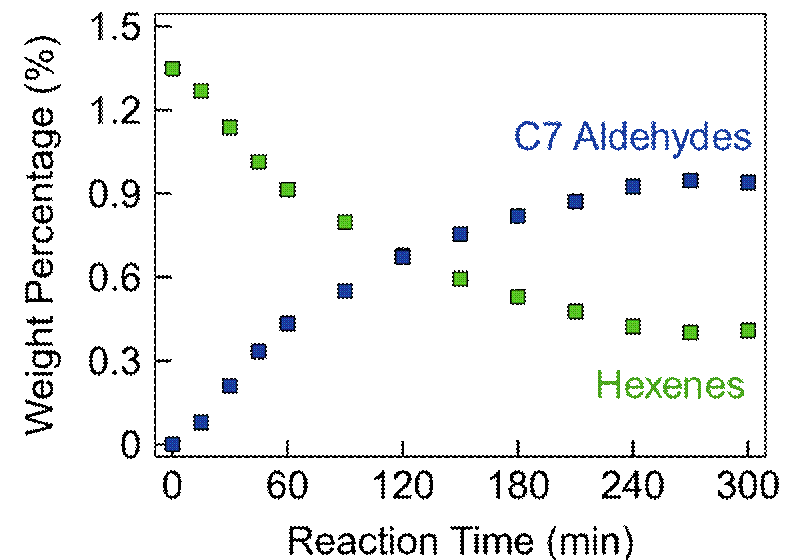


**Figure S4.** Hydroformylation of 1-hexene with unmodified Co_2_(CO)_8_ catalyst.

We first tested hydroformylation with 1-hexene and surfactant-range model pyrolysis oil (1:1 ratio of dodecene and hexane).

As shown in **Figure S5a**, complete conversion of olefins was achieved in both scenarios as evidenced by the disappearance of double bonded carbon peaks within the range of 110–150 ppm in ^13^C NMR spectra. The majority of dodecene was converted to aldehydes leading to peaks in the range of 200–210 ppm after 120 min. The appearance of the peaks in the range of 60–70 ppm indicates the formation of alcohols, which increased while aldehyde production decreased in the 300 mins spectrum. If alcohols are the targeted product, then a tandem hydroformylation-hydrogenation reactor for converting olefins to primary alcohols would be desirable.^[5]^

All NMR experiments were conducted on Bruker Avance-500 with a DCH cryoprobe at University of Wisconsin-Madison Chemistry NMR Facility. The sample was dispersed in CDCl_3_ (sample/CDCl_3_=1/7 mass ratio), sonicated, and transferred to an NMR sample tube. The parameters for NMR experiments are shown below:

1) ^1^H NMR (zg30): 32 scans, delay of 1 s, 90-degree pulse width of 9.6 µs

2) ^13^C NMR (zgig): 80 scans, delay of 20 s, 90-degree pulse width of 10.35 µs

3) HSQC (hsqcedetgp): the acquire size was [1024, 400] with 2 scans per increment, delay of 2s.


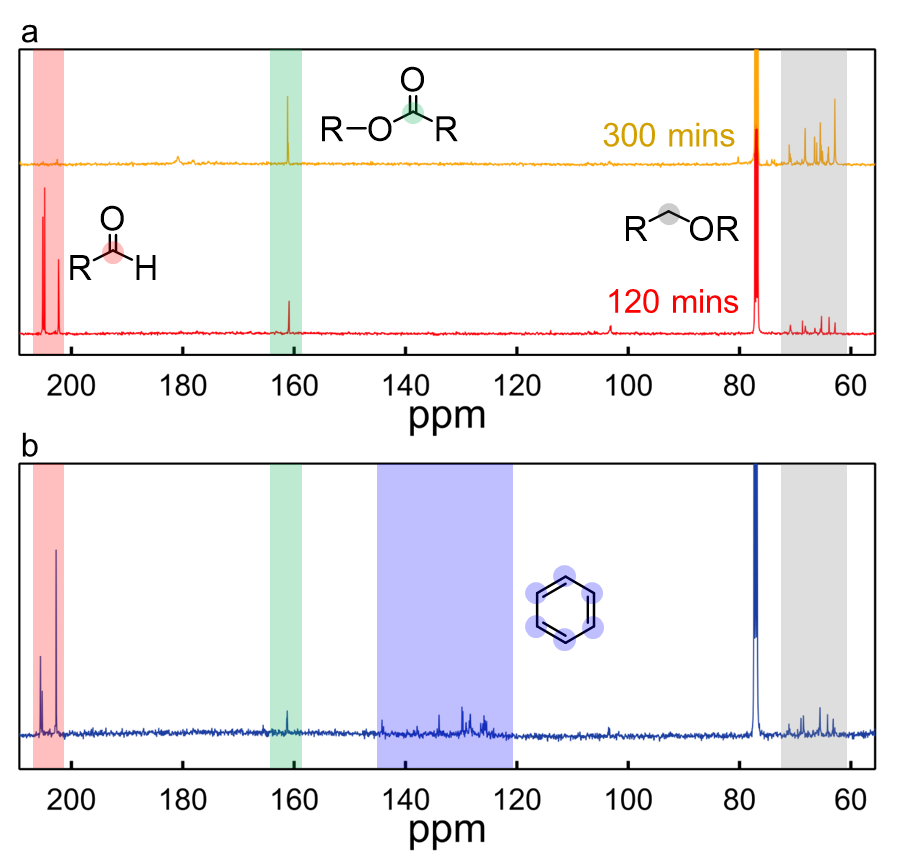


**Figure S5.** ^13^C NMR spectra of hydroformylated (a) surfactant-range model pyrolysis oil and (b) surfactant-range pyrolysis oil catalyzed by unmodified Co_2_(CO)_8_ catalyst. CDCl_3_ was used as a solvent for NMR measurement. R in the structural formula represents organic groups or hydrogen.

Pinnick oxidation begins with the aldehyde undergoing nucleophilic attack by the hypochlorite ion (ClO⁻), forming a chloro-hydroxy intermediate which then undergoes intramolecular rearrangement generating carboxylic acids (**Scheme S1**).^[6]^ H_2_O_2_ is also required to neutralize excess hypochlorous acid, thus preventing over-oxidation or chlorination. The side products generated, such as HCl, O_2_, and H_2_O, can be easily removed from the oxidized oil by taking advantage of the drastic differences in polarity between these species and target carboxylic acids. In a typical experiment, acetonitrile, hydrogen peroxide, potassium phosphate, and water were combined in a round bottom flask in that order. After cooling in an ice bath, a 14.5 wt.% solution of sodium chlorite in water was added dropwise to the flask. The mixture was stirred vigorously in a fume hood for at least eight hours, after which the aldehydes had been fully oxidized into carboxylic acids. The mixture was then quenched with sodium sulfite followed by acidification with a 37 wt.% hydrochloric acid in water solution. Following oxidation, liquid-liquid extraction with dichloromethane (DCM) allowed for the removal of carboxylic acids into DCM followed by drying with magnesium sulfate and rotary evaporation leaving the final product as oxidized oil.


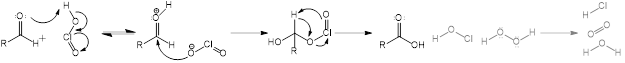


**Scheme S1**. Mechanism of Pinnick oxidation followed by scavenging with H_2_O_2_.

It is challenging to determine and present the product composition due to the multiple solvents that were used. Thus, ^1^H NMR was used to analyze the products after Pinnick oxidation as shown in **Figure S6**. The peaks within the range of 2.2–2.5 ppm in the red curve of **Figure S6** correspond to the alpha protons of the carboxyl or carboxylate groups in the oxidized products from hydroformylated dodecene. In addition, the peaks at 3.4–3.6 ppm and 4.1–4.3 ppm are attributed to protons associated with carbons bonded to oxygen in the hydroxyl and carboxyl groups, respectively. The ratio of the areas of these peaks represents the molar ratio of species in the product, indicating the formation of carboxylic acids from aldehydes, while alcohols and esters remain after the oxidation step. A similar phenomenon was observed using surfactant-range hydroformylated oil as exhibited in the blue curve of **Figure S6**. Considering that protons in both carboxylic acids and esters contribute to the peaks in the range of 2.2–2.5 ppm, while only protons in esters contribute to the peaks at 4.1–4.3 ppm, subtracting the area of the latter peaks (4.1–4.3 ppm) from the area of the former peaks (2.2–2.5 ppm) in the blue curve gives the amount of protons bonded to the carbon adjacent to the carboxyl group carbon. Therefore, the molar percentages of carboxylic acids, alcohols, and esters can be determined; the percentages are similar to the molar percentages of aldehydes, alcohols, and esters in the surfactant-range hydroformylated oil discussed in the previous paragraph. This result suggests that Pinnick oxidation obtains near-quantitative conversion of the aldehydes to carboxylic acids after hydroformylating the plastic pyrolysis oil.


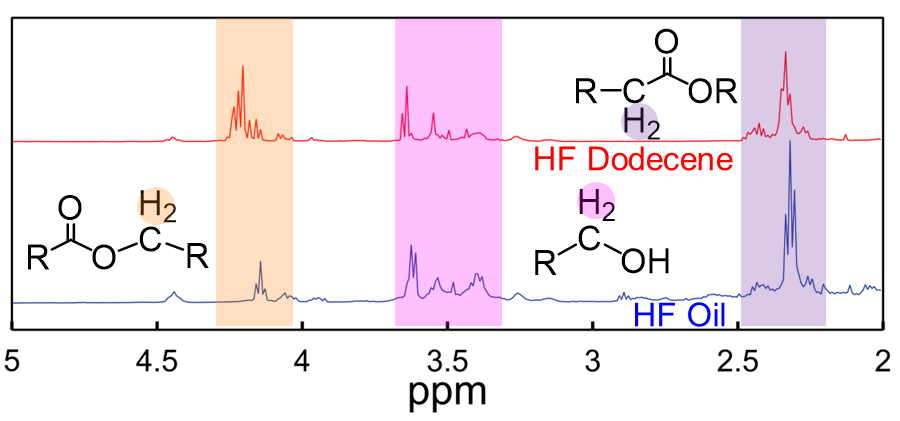


**Figure S6.** ^1^H NMR spectra of oxidized hydroformylated dodecene (red) and hydroformylated surfactant-range pyrolysis oil (blue) via Pinnick oxidation. CDCl_3_ was used as a solvent for NMR measurement. R in the structural formula represents organic groups or hydrogen.

NMR observes functionalities rather than separating by size or polarity. With NMR we can conclude that Pinnick oxidation had near complete conversion of aldehydes. As shown by the disappearance of the formyl peak (red) and appearance of acid peaks (green) from ^13^C NMR in **Figure S7**.


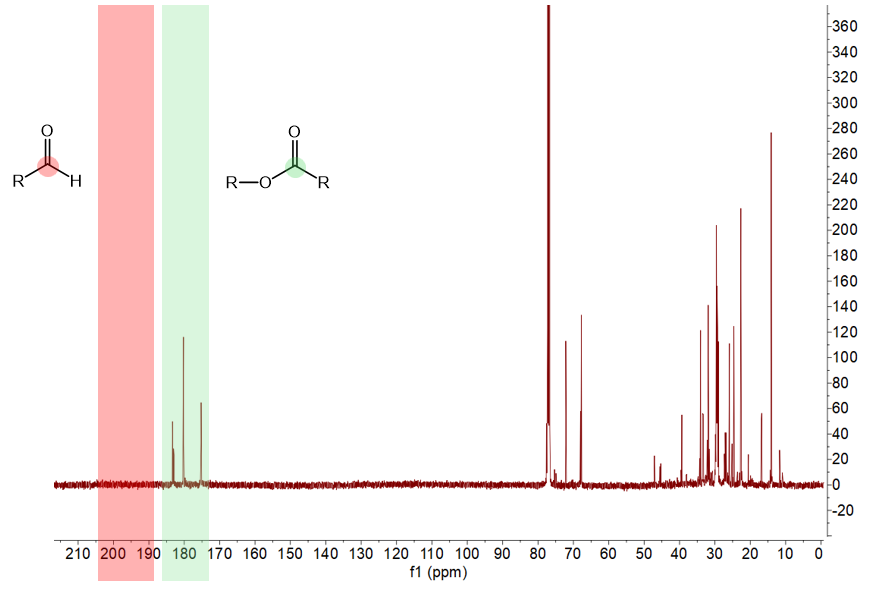


**Figure S7.** ^13^C NMR spectra of oxidized pyrolysis oil. CDCl_3_ was used as the NMR solvent. R represent carbon groups or hydrogen.

An analytical technique is needed to quantitatively determine carboxylic acid formation after Pinnick oxidation. Typically, these acid groups would damage GC columns, especially those with capillary films. Here N-methyl-N-(trimethylsilyl)trifluoroacetamide (MSTFA) is used as a silylation agent to prevent this corrosion (**Scheme S2**).^[7]^ Using this agent does require a unique calibration curve to be developed as the derivatized acids will have unique response factors but allows for the oxidation of model compounds (dodecanoic acid) to be studied to validate complete aldehyde conversion. Once the proper conditions were found for model compound oxidation, the conditions were applied to hydroformylated pyrolysis oil with the assumption of high conversion. This assumption was verified using ^13^C NMR (**Figure S7**).


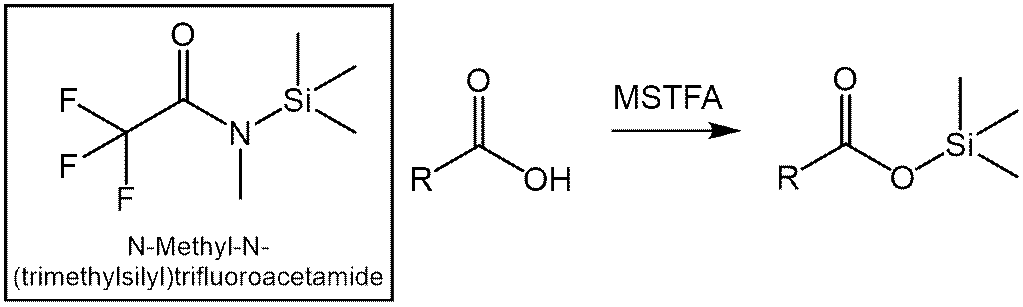


**Scheme S2**. Derivatization of carboxylic acid using MSTFA.

Samples after hydroformylation and oxidation were quantified by GC-FID (Shimadzu, Nexis GC 2030) and identified with GC-MS (Shimadzu, GC 2010). Both GCs had a 30 m Restek RTX-VMS column installed. For each measurement, the oven was initially set to 40 °C and held for 5 min. Then with a ramping rate of 5 ℃/min the oven was heated to 100 °C. The temperature was then increased to 240 ℃ with a ramping rate of 20 °C /min and held at 240 ℃ for another 11 min. GC-MS was used to analyze the products in the oxidized surfactant-range oil, as depicted in **Figure S8**. This figure also illustrates the difficulty of interpreting 1D GC data with these plastic derived samples. MSTFA was used as a silylation reagent for the derivatization of carboxylic acids in the oxidized oil to increase their volatility and stability in gas chromatography (**Scheme S2**). Both aldehydes and carboxylic acids are derivatized by MSTFA. The absence of aldehyde derivatives suggests the complete conversion of aldehydes to carboxylic acids, consistent with the NMR results (**Figure S6**). More than 20 organooxygen compounds were identified between C10 and C15 (excluding the trimethylsilyl group), with the major product series exhibiting molecular formulas of C_x_H_y_O_2_, highlighted in red in **Figure S8**, and primarily consisting of carboxylic acids with varying degrees of branching.


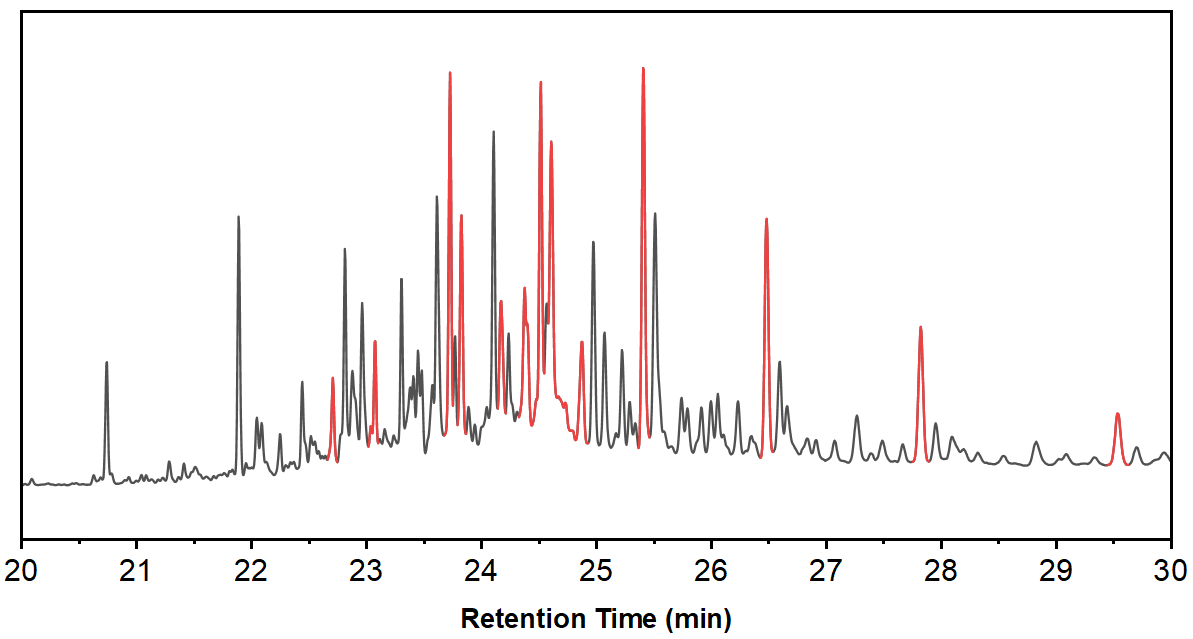


**Figure S8.** GC-MS of oxidized surfactant-range oil after MSTFA derivatization. Peaks corresponding to derivatized carboxylic acids are highlighted in red.

With GC-FID combined with GC-MS, identification of acid peaks (**Table S5**) was performed to confirm that surfactant range pyrolysis oil had been chemically converted. Both GCs have a 30 m Restek MXT-1HT column installed. With the same column installed the retention times and peak placement of two spectra can be compared across each instrument. However, due to the complexity of the oil it is challenging to fully separate all compounds with one dimensional GC. Possibly, overlapped acid peaks have been missed in this analysis. This analysis serves as proof of the presence of surfactant-range acids not as quantification of concentration, e.g. the average molecular weight of the acids is unknown.

**Table S5.** GC-MS and GC-FID results to predict carboxylic acids present in oxidized oil.

| **GC-MS Retention Time (min)** | **Chemical Name** |
| --- | --- |
| 22.66 – 22.75 | Octanoic Acid |
| 23.01 – 23.1 | Branched Octanoic Acid |
| 23.68 – 23.73 | Branched Nonanoic Acid |
| 23.79 – 23.87 | Branched Nonanoic Acid |
| 24.17 – 24.21 | Branched Nonanoic Acid |
| 24.33 – 24.44 | Nonanoic Acid |
| 24.44 – 24.55 | Branched Nonanoic Acid |
| 24.60 – 24.82 | Branched Decanoic Acid |
| 24.82 – 24.93 | Branched Decanoic Acid |
| 25.37 – 25.46 | Decanoic Acid |
| 26.43 – 26.54 | Undecanoic Acid |
| 27.75 – 27.89 | Dodecanoic Acid |
| 29.45 – 29.63 | Tridecanoic Acid |

Hydroformylation of C9–C14 olefins produces alcohols in addition to aldehydes. This undesired byproduct that must either be removed or chemically converted. For this reason, we designed a process (**Figure 2** from main text) that can both acidify these alcohols and remove the paraffins and aromatics present in pyrolysis oil. After Pinnick oxidation, this oil was mixed with anhydrous tetrahydrofuran (THF) in another round bottom flask for the acidification of alcohols and the removal of undesirable species. THF must be anhydrous as experiments with trace amounts of water in the THF had little to no conversion of the alcohol to the acid because sodium hydride reacts with water. The mass ratio of oil to THF was 0.15:1 to fully dissolve the oil. Dodecanol was used as a model compound for the acidification of alcohols in the oxidized oil, representing the average length of a surfactant-range alcohol. Sodium hydride (2.5 equiv.) was slowly added to salt the alcohol and carboxylic acid groups. Sodium hydroxide was tested as a safer substitute for sodium hydride. However, NaOH lacked reactivity with dodecanol. The pyrophoric nature of sodium hydride requires this reaction to be done in an ice bath. Iodine (0.08 equiv.) was then added into solution under an ice bath, reacting with sodium hydride to produce sodium iodine, a nucleophilic catalyst for this reaction. SMCA (2.5 equiv.) was then added, substituting a carboxymethyl group onto the salted alcohols. SMCA did not fully dissolve in THF causing mass transfer limitations. For this reason, small volumes of polar solvents such as HMPA were added to the THF/oil mixture, quantities shown in **Table S6**. The ice bath was removed after SMCA was added followed by heating the flask in a silicone oil bath to 60 ℃. After 24 hours of constant stirring, the liquid was slowly quenched with water. Next, all paraffins and aromatics were extracted through a liquid-liquid extraction using diethyl ether. After acidifying the aqueous phase to a pH of 1 with sulfuric acid, carboxylic acid groups were then extracted to a nonpolar DCM phase. Drying with magnesium sulfate and removing the DCM through rotary evaporation (30 ℃ and 700 mbar) results in a product consisting only of carboxylic acids.

Multiple iterations of this process were tested with the results leading to a higher degree of conversion shown in **Table S6**. The two most substantial improvements came from the addition of HMPA and the grinding of SMCA. HMPA proved difficult to remove, so longer time spans with ground SMCA were preferred for the separation of oxidized plastic pyrolysis oil. The oxidized oil sample was left reacting for five days to ensure complete acidification of alcohols. HMPA could not be completely removed through liquid-liquid extractions with diethyl ether and DCM. Grinding the SMCA increased the yield of 2-(dodecyloxy)acetic acid even in the absence of HMPA. Without HMPA and by extending the reaction time, we were able to achieve 99% conversion of dodecanol.

**Table S6.** Alcohol acidification yields. *SMCA was ground. **SMCA was ground while acids and paraffins were introduced.

| **Time (h)** | **Volume HMPA (mL)** | **Conversion to 2-(dodecyloxy)acetic acid (%)** |
| --- | --- | --- |
| 24 | 0 | 66 |
| 72 | 0 | 70 |
| 24 | 4 | 97 |
| 24* | 0 | 82 |
| 72* | 4 | 99 |
| 72** | 4 | 99 |
| 96** | 0 | 99 |

**Figure S9a** indicates the successful acidification and purification of our oil with no alcohol or aldehyde peaks, which appear at 2 – 2.5 ppm for the protons near alcohol groups and 9 – 10 ppm for the aldehyde protons. The peak present at 2.3 ppm stems from a carboxylic acid group rather than an alcohol as confirmed by the peak splitting 2D NMR. Only methyl, ethyl, and solvent peaks are present outside of the ppm range for carboxylic acids (shown in **Figure S9b**) demonstrating that a high purity of carboxylic acids are present for the surfactant analysis.

**
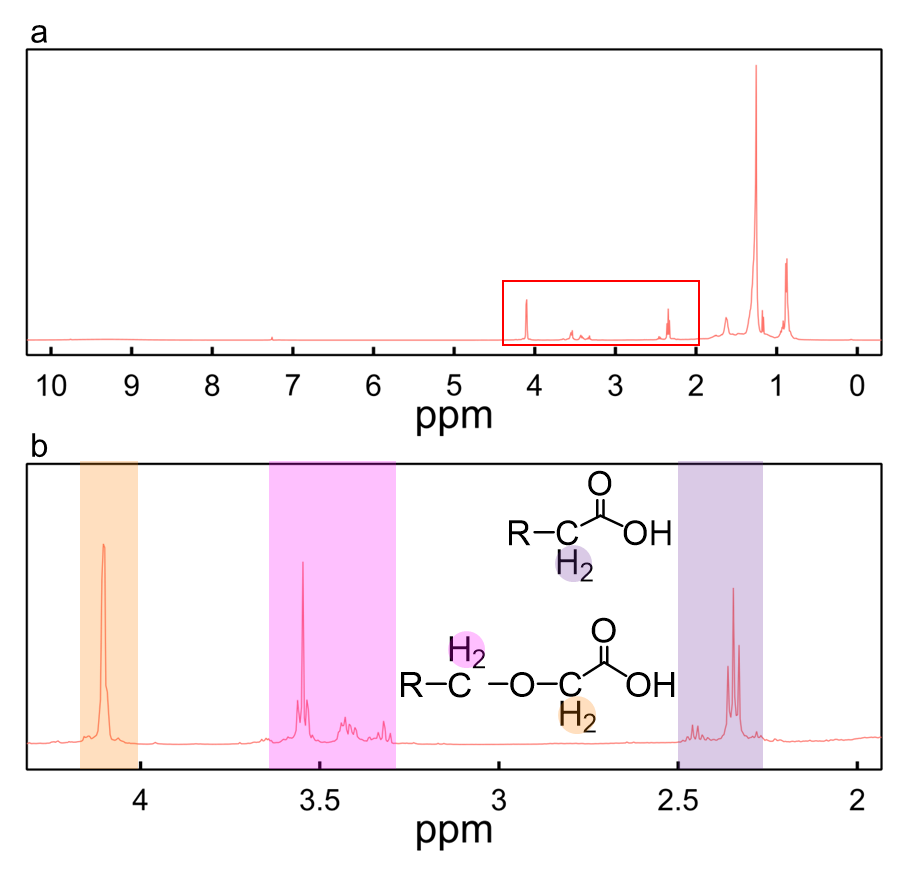
**

**Figure S9**. ^1^H NMR (a) full spectrum and (b) magnified boxed region in full spectrum of PDCs after acidification and purification. CDCl_3_ was used as a solvent for NMR measurement. R in the structural formula represents organic groups or hydrogen.

***Surfactancy measurements***

For all surfactancy testing, the carboxylic acid oil was neutralized with NaOH after dispersion of the former in water and dissolution of the latter in the same water forming the final plastic-derived carboxylate surfactant (PDC) product. All water used was ultra-pure distilled (DI) water (resistance = 19.8 MΩ·cm) from a Millipore Synergy Water Purification System, ensuring consistency in experimental conditions. Using the assumption described earlier that the carbon-chain length was 12 carbons and that the oil was pure carboxylic acid, the mass ratio of the plastic-derived oil to NaOH was 5.2:1. After neutralization, plastic-derived carboxylate surfactants (PDCs) were formed, and no oil was visibly present. This material (10 wt% surfactant in water) was used as the stock solution for subsequent characterization experiments.

The critical micelle concentration (CMC) was determined in DI water using surface tension measurements with a DataPhysics DCAT surface tensiometer and a platinum Wilhelmy plate that was cleaned via a natural gas flame. Prior to measurement, 30 mL of distilled water was equilibrated to 25°C in a 70 mL vessel using a temperature-controlled water bath. Surfactant solutions of known concentrations were introduced via a syringe pump. After each surfactant addition, the solution was mixed and allowed to rest for one minute before measuring surface tension. If the standard deviation of the surface tension remained within ±0.005 mN/m for the final 60 seconds of a 6-minute measurement, the average value over this period was recorded. If this criterion was not met within 20 minutes, the average surface tension from 19 to 20 minutes was used. The CMC was determined by fitting a line to user-selected high- and low-concentration ranges of data points, with the intersection of the two lines representing the CMC.

Foam analysis of surfactants was conducted at room temperature using a Krüss Dynamic Foam Analyzer-100 with ADVANCE software. A 1 wt% surfactant solution in deionized water was prepared using a magnetic stirrer to ensure complete mixing, followed by a 10-minute resting period before measurement. Foam was generated in a 250 mm-high, 40 mm-diameter prism column filled with the surfactant solution by sparging air through a porous filter plate (40–100 µm pore size) at a controlled gas flow rate of 0.2 L/min. Foam formation was halted after 30 seconds. An optical sensor measured the foam volume, while seven conductivity sensors positioned along the column monitored liquid content and resistance at different heights and one chip on the bottom as the reference. A camera (2 fps at 1280 × 1024 px), set at 55 mm height, captured images and videos at various time intervals up to a maximum of 3600 seconds.

Dynamic surface tension measurements were conducted using a Krüss BP100 bubble pressure tensiometer at a surfactant concentration of 0.5*CMC. Hydrophobic ally modified glass capillary with an inner diameter of 0.228 mm was used for the measurements. Surface ages of the bubbles varied from 10 ms to 10 s. The diffusion coefficient was calculated using the Ward & Toradi model shown in **Equation S1**:

$$\begin{aligned} \gamma_{e}=\gamma_{eq}+\frac{2nRT\Gamma_{eq}^{2}}{c}\sqrt{\frac{\pi}{4Dt}}\#\left( S1 \right) \end{aligned}$$

Where:

- γ_e_ = Surface tension at time t (N/m)
- γ_eq_ = Equilibrium surface tension (N/m)
- n = Adsorption constant (equals 2 for ionic surfactants)
- R = Universal gas constant (8.314 J/(mol·K))
- T = Absolute temperature (K)
- Γ_eq_= Equilibrium surface excess concentration (mol/m²)
- C = Bulk surfactant concentration (mol/m³)
- D = Diffusion coefficient of the surfactant (m²/s)
- t = Time (s)

Γ_eq_ was calculated using **Equation S2** from the linear part of the surface tension vs. log C below the CMC:

$$\begin{aligned} \Gamma_{eq}=-\frac{1}{nRT}\left( \frac{d\gamma}{dlnC} \right)\#\left( S2 \right) \end{aligned}$$

To measure calcium tolerance of the surfactants, a method developed by Procter and Gamble was deployed. A mixture containing 450 ppm surfactant and 420 ppm cation chloride solution (3:1 Ca2^+^: Mg^2+^) was prepared and left overnight at room temperature. The supernatant was extracted and filtered by a 0.22 μm filter and then the surfactant concentration (which is the calcium tolerance) after precipitation was determined by UV/Vis at 210 nm. Surfactant solutions with concentrations ranging from 0.01 wt% to 0.1 wt% were prepared to establish a calibration curve relating absorbance to concentration. This calibration curve was then used to determine the concentration of the unknown surfactant solution. Calcium tolerance is a critical parameter for evaluating the robustness of anionic surfactants and colloidal systems in hard water conditions or industrial formulations, where divalent ions such as calcium and magnesium can significantly alter adsorption behavior and stability.

Small-angle X-ray scattering (SAXS) measurements were performed using a Xenocs XEUSS 3.0 with Cu-K-alpha radiation (wavelength of 1.54 Å). The apparatus was operated at 50 kV and 0.6 mA with a q-range of 0.01 to 0.3 Å^-1^ at room temperature. Samples were filled in an 80-mm-long quartz capillary with an outside diameter and wall thickness of 1.5 and 0.01 mm, respectively. Exposure time was set to 480 minutes. Scattering data was acquired using an image plate detector, producing a two-dimensional scattering pattern. This 2D pattern was processed with XSACT software to obtain a one-dimensional I(q) profile, where intensity (I) is plotted as a function of the scattering vector (q). Background scattering was measured separately and background contributions from the DI water solvent and capillary were subtracted to isolate the sample signal using standard background subtraction protocols.

To justify the statement made in the main text that the higher molecular weight, e.g. lower CMC, components dominate the CMC of the mixture, Rubingh’s equation, which utilizes regular solution theory, has been used extensively to predict the CMC, for surfactant mixtures.^[8]^ **Equation S3** shows the expression:

$X_{1}{*CMC}_{12}=Y_{1}{CMC}_{1}e^{\beta\left( 1-Y_{1} \right)^{2}}$(S3)

$\left( 1-X_{1} \right)*{CMC}_{12}={(1-Y}_{1}){CMC}_{2}e^{\beta\left( Y_{1} \right)^{2}}$ (S4)

Where X_1_ and X_2_ are the mole fractions of 1 and 2 in bulk and Y_1_ and Y_2_ are mole fraction of 1 and 2 in the micelle. β is termed the interaction parameter and is defined in terms of the excess enthalpy (ΔH_e_) or free energy (ΔG_e_) of mixing in the micelle.

$\Delta H_{e}=\Delta G_{e}=Y_{1}Y_{2}\beta RT$ (S5)

Negative β, which is typically the case, means synergistic mixing and there is some alternating character to the arrangement of micelles and the micelle will be enriched in the minor component.

For the case of homologous charged surfactants in water with no electrolyte that differ only in chain length, an empirical expression was developed showing that for Δn_c_<0.75 (n_c_ is the number of carbon atoms on the hydrophile) the mixture is ideal, for 0.75<Δn_c_<5.31, δβ/δΔn_c_=-0.53 while 5.31< Δn_c_ δβ/δΔn_c_= -1.43.^[8]^ A negative value is consistent with synergistic mixing; synergy occurs in homologous series because head group repulsion is reduced relative to the single-component surfactants. **Figure S10** shows the CMC as a function of mole fraction for a mixture of SDC (C10) and sodium myristate (C14) according to **Equations S1 and S2** and β=-2.12 (value calculated for Δn_c_=4) with the values for the CMC given in the main paper. **Figure S10** shows that the CMC of the mixture is dominated by the CMC of the lower CMC component; such an observation is always true for synergistic mixtures.

**
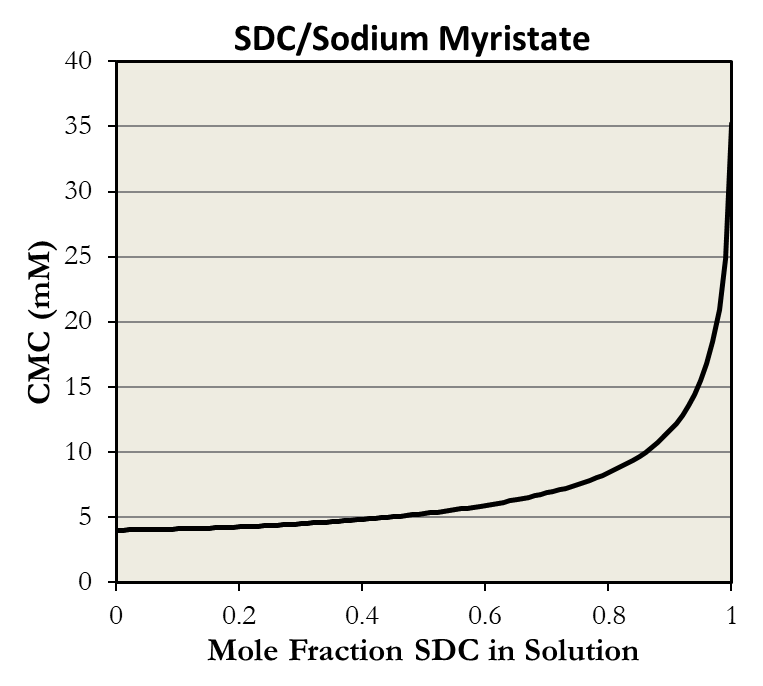
**

**Figure S10.** CMC prediction for a mixture of sodium myristate with SDC.

No evidence of precipitation at or below 10 wt% was found for PDC; higher concentrations were not tested. Meanwhile, $\bar{M}\bar{B}\bar{A}$ refers to the average projected 2D area of individual bubbles within the foam structure over time. The foam generated by SLC exhibited a half-life exceeding 3600 seconds, while the foam produced by PDC had a half-life of exactly 3600 seconds. Meanwhile, foam from SDC showed a much shorter half-life of 1695 seconds. For all three surfactants the drainage half-life time exceeds 3600 seconds. Additionally, the initial $\bar{M}\bar{B}\bar{A}$ of PDC foams was between those observed for SDC and SLC. At the final stage, $\bar{M}\bar{B}\bar{A}$ for PDC was higher than that of SLC, while no final $\bar{M}\bar{B}\bar{A}$ was recorded for SDC because all of the bubbles had disappeared after 3600 seconds. The higher $\bar{M}\bar{B}\bar{A}$ value of PDC further supports the conclusion that PDC exhibits greater foamability relative to SLC and SDC. Furthermore, PDC foams exhibited a lower bubble count overall, indicating the presence of larger, more stable bubbles compared to SLC.

**Figure S11** presents the dynamic surface tension profiles of SDC, SLC, and PDC at 0.5*CMC measured using a bubble pressure tensiometer as described previously. The surface tension decreases with increasing bubble surface age due to the time-dependent adsorption of surfactant molecules at the air–water interface. At short times, the adsorption of all surfactants is diffusion limited, consistent with predictions from the Ward & Tordai model, **Equation S1.** Note that 0.5*CMC is in the linear region of the log (surface tension) vs. surfactant concentration plot (**Figure 3** from the main text) and hence using **Equation S2** to determine Γeq for the Ward & Tordai is entirely appropriate.


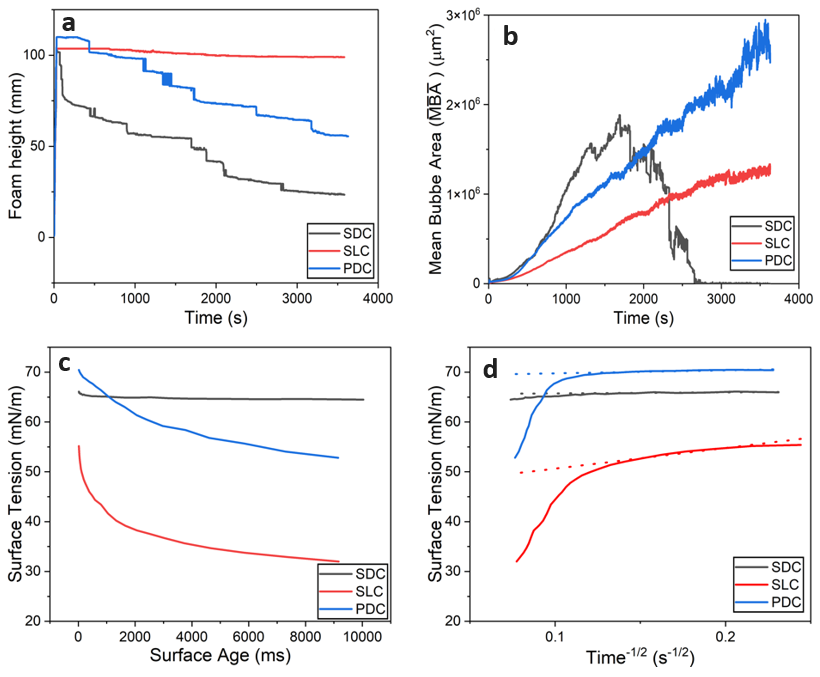


**Figure S11.** a) Height decay profile of the surfactant, b) Mean bubble area as a function of time for the surfactants, Plot of dynamic surface tension of surfactants c) as a function of surface age and d) as a function of time^-1/2^. Dotted lines represent the fitted model at short times.

For SAXS measurements, SDC and SLC were used at concentrations of 10 times their respective CMC. Initially, PDC was also tested at 10×CMC; however, the resulting signal was too weak due to PDC's CMC being approximately one order of magnitude lower than that of SDC and SLC, leading to a much lower absolute concentration. To overcome this, the PDC concentration was increased to 4 wt%, which yielded a sufficiently strong signal for reliable model fitting. q_max_ (e.g. q where scattered intensity is a maximum) depends on the length of the hydrophobic alkyl chain, with longer chains resulting in lower q_max_.^[9]^ Specifically, q_max_ values for SDC, SLC, and PDC were determined to be 0.21, 0.17, and 0.16 cm^-1^, respectively, consistent with PDC containing longer-chain components.

**
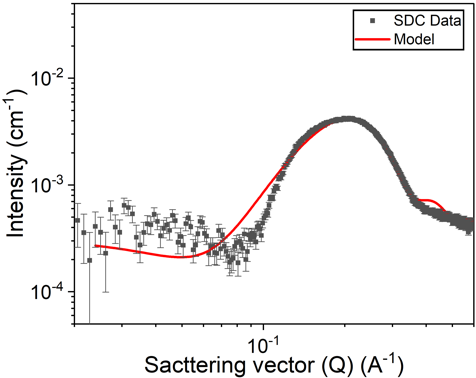

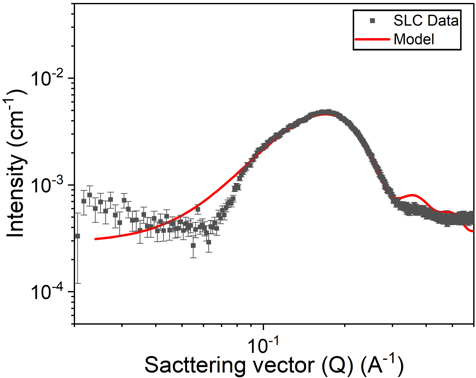

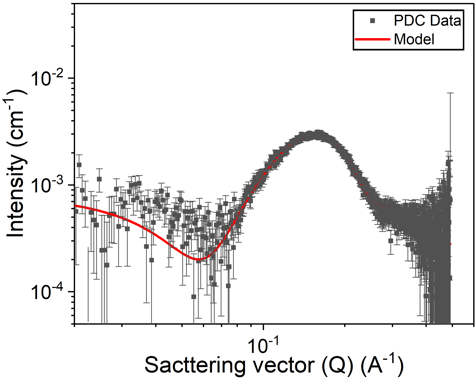
**

**Figure S12.** Data and fit for SAXS pattern for SDC, SLC and PDC.

Data was fit to morphological models using SasView software. A micelle exhibits three distinct electron density regions: the core, the water, and the micellar shell. During fitting, the electron density of water was fixed at 334 electrons per cubic nanometer (e⁻/nm³). The procedure for model fitting was to start with the simplest model (e.g. a monodisperse distribution of core_shell_spheres) and then increase the complexity until the fits were good and the parameters (core thickness, shell thickness and their respective electron densities) were reasonable. Other than a monodisperse distribution of spheres, a polydisperse distribution of core_shell_spheres was also tested. Using the criteria above, acceptable fits for all the data sets were accomplished with the core_shell_ellipsoid model with all micelles being slightly oblate ellipsoids. Including polydispersity in this model did not dramatically increase the fit quality, as quantified by the fitting error (Χ^2^), therefore the monodisperse version of the model was used. Graphs of the data and the best fit model are shown in **Figure S12** and model parameters for the best fits are summarized in **Table S7**. The fits to the PDC samples are much better than the fits to the SLC and SDC samples according to the Χ^2^. A denser shell compared to the core is an indication of a narrower particle size distribution.^[10]^ Based on the best fit values, the hydrocarbon core of the PDC is ~25% less electron dense than those of the SLC or SDC while the shells are ~25% more electron dense than those for the SLC or SDC. Such a result was not expected, but we do not feel that the result is unreasonable, especially given the inherent uncertainty in choosing the correct model. Further, the reduction in electron density of the core could indicate a lower core packing density due to the more random hydrophobic chain morphologies expected in the PDC samples when compared to the ~monodisperse carbon chain length SDC or SLC surfactants. To our knowledge the effect of polydispersity on the electron density of the core has not been reported in the literature. According to the equatorial radius (R), SDC micelles are smaller than the SLC micelles, while both are smaller than the PDC micelles as expected according to q_max_. As expected, thicknesses of the shell/head group layer (s) are similar among the different surfactant species and are likely within experimental error. Finally, the aspect ratios were found to be consistent within experimental error, and within expectations based on other literature studies.^[11–13]^

**Table S7.** Fitting parameter obtained from the fits.

|  | R | Aspect  Ratio | s | Scattering Length Density | | | Electron Density | | | Χ^2^ |
| --- | --- | --- | --- | --- | --- | --- | --- | --- | --- | --- |
|  |  |  |  | Core | Shell | Solvent | Core | Shell | Solvent |  |
|  | Å | - | Å | 10^-6^/ Å^2^ | 10^-6^/ Å^2^ | 10^-6^/ Å^2^ | e^-^/ nm^3^ | e^-^/ nm^3^ | e^-^/ nm^3^ | - |
| SDC | 16.56 | 0.62 | 4.04 | 7.77 | 11.10 | 9.5 | 273 | 390 | 334 | 13.06 |
| SLC | 19.84 | 0.65 | 3.79 | 7.13 | 12.29 | 9.5 | 251 | 432 | 334 | 15.43 |
| PDC | 23.69 | 0.61 | 4.43 | 5.21 | 15.53 | 9.5 | 183 | 568 | 334 | 1.45 |

**References:**

[1] H. Li, J. Wu, Z. Jiang, J. Ma, V. M. Zavala, C. R. Landis, M. Mavrikakis, G. W. Huber, *Science* **2023**, *381*, 660–666.

[2] J. Wu, Z. Jiang, V. S. Cecon, G. Curtzwiler, K. Vorst, M. Mavrikakis, G. W. Huber, *Green Chem.* **2024**, *26*, 11908–11923.

[3] H. K. Abdel-Aal, G. Al-Shaikh, in *Pet. Econ. Eng.*, CRC Press, **2013**.

[4] T. Liu, L. Yang, H. Jiao, Z. Jin, P. Chen, S. Leng, W. Zhou, *Algal Res.* **2022**, *68*, 102888.

[5] K. Takahashi, M. Yamashita, K. Nozaki, *J. Am. Chem. Soc.* **2012**, *134*, 18746–18757.

[6] F. Foarta, C. R. Landis, *J. Org. Chem.* **2016**, *81*, 11250–11255.

[7] J. L. Little, *J. Chromatogr. A* **1999**, *844*, 1–22.

[8] B. P. Grady, *J. Surfactants Deterg.* **2023**, *26*, 237–250.

[9] A. Bhadani, T. Okano, T. Ogura, T. Misono, K. Sakai, M. Abe, H. Sakai, *Colloids Surf. Physicochem. Eng. Asp.* **2016**, *494*, 147–155.

[10] L. A. Fielding, O. O. Mykhaylyk, S. P. Armes, P. W. Fowler, V. Mittal, S. Fitzpatrick, *Langmuir* **2012**, *28*, 2536–2544.

[11] “Synthesis and Characterization of Novel Surfactants Based on 2‐Hydroxy‐4‐(methylthio)butanoic Acid: 2. Non‐ionic Surfactants - Yu - 2017 - Journal of Surfactants and Detergents - Wiley Online Library,” can be found under https://aocs.onlinelibrary.wiley.com/doi/abs/10.1007/s11743-016-1915-7, **n.d.**

[12] R. C. Oliver, J. Lipfert, D. A. Fox, R. H. Lo, S. Doniach, L. Columbus, *PLOS ONE* **2013**, *8*, e62488.

[13] J. Lipfert, L. Columbus, V. B. Chu, S. A. Lesley, S. Doniach, *J. Phys. Chem. B* **2007**, *111*, 12427–12438.
